# A Comprehensive Review on the Effects of Humor in Patients With Depression

**DOI:** 10.7759/cureus.29263

**Published:** 2022-09-17

**Authors:** Oghenetega E Ayisire, Funmilola Babalola, Bialo Aladum, Oluwabukola C Oyeleye-Adegbite, Alexsandra Urhi, Akinkunmi Kilanko, Chukwudi Agbor, Ngozi Adaralegbe, Garima Kaur, Chioma Eze-Njoku, Fareena Soomro, Victor C Eche, Hakeem A Popoola, Gibson O Anugwom

**Affiliations:** 1 Psychiatry, University of South Wales, Pontypridd, GBR; 2 Epidemiology and Public Health, Texas Department of State Health Services, San Antonio, USA; 3 Psychiatry, Ascension Borgess Hospital, Kalamazoo, USA; 4 Department of Public Health, Texas A&M University, College Station, USA; 5 Department of Psychiatry, Federal Neuro Psychiatric Hospital, Benin City, NGA; 6 Behavioral Sciences, La Sierra University, Riverside, USA; 7 Psychiatry, Tees, Esk and Wear Valleys NHS Trust, Scarborough, GBR; 8 Psychiatry and Behavioral Sciences, University of Connecticut, Waterbury, USA; 9 Psychiatry, All India Institute of Medical Sciences Rishikesh, Rishikesh, IND; 10 Pediatrics, Atrium Health Navicent Medical Center, Macon, USA; 11 Psychiatry and Behavioral Sciences, Isra University, Sindh, PAK; 12 Psychiatry, University of Port Harcourt, Port Harcourt, NGA; 13 Internal Medicine, Windsor University School of Medicine, Basseterre, KNA; 14 Menninger Department of Psychiatry and Behavioral Sciences, Baylor College of Medicine, Houston, USA

**Keywords:** humor therapy, humor-style, relationship, depression, humor

## Abstract

Depression is a leading cause of disability worldwide and a major contributor to the overall global burden of disease. Although there are known, effective treatments for depression, people in low- and middle-income areas experience multiple barriers which limit their ability to receive adequate treatment. Some known barriers to effective care include a lack of resources, lack of trained healthcare providers, and social stigma associated with mental disorders and this creates gaps in mental health care and the need for more treatment modalities and adjuvant therapies to address these gaps. This review article was conducted using the scale for the assessment of non-systematic review articles (SANRA). We searched three databases; EMBASE, PubMed (MEDLINE), and Google Scholar using specified search terms. We had a total of nine articles with sample sizes ranging from 37 to 1551, and the age of participants ranged from 23 to 93 years. Articles were diverse in race and geographical locations. The articles were derived from cross-sectional studies, randomized studies, and experimental studies, and they focused on the relationship between humor and depression, and the reduced risk of depression in the study population. The articles identified different aspects of the relationship between humor and depression. The willingness of patients with depression to recognize or participate in humor could be defective resulting in abnormal social interactions such as withdrawal. However, there was some significant influence of humor or its styles on patients with depression either mitigating depressive symptoms or having no impact at all.

## Introduction and background

Globally, it is estimated that 5% of adults suffer from depression, a leading cause of disability worldwide and a significant contributor to the global burden of disease [1]. Although there are known effective treatments for depression, more than 75% of people in low- and middle-income areas receive inadequate treatment [2]. Barriers to delivering effective care to this patient population include a lack of resources, a lack of trained healthcare providers, and poor health-seeking behaviors due to social stigma associated with mental disorders [1]. It has created significant mental health care gaps that necessitate better strategic treatment modalities and adjuvant therapies to address these gaps. Humor has been a focus of attention in the medical literature. However, despite statements about its health benefits, research is insufficient to validate such claims [3]. Earlier studies about the relationship between depression and humor showed improved symptoms and quality of life among depressed study participants [4]. Hence, this review aims to explore the relationship between humor and depression, identify the effects of humor on patients with depression, and point out possible gaps for future research. 

## Review

Methodology

Search Strategy 

This review article was conducted using the scale for the assessment of non-systematic review articles (SANRA). We searched three databases; EMBASE, PubMed (MEDLINE), and Google Scholar using specific search terms. Search terms on the Medline and EMBASE database used were “effects of humor” AND “depression”. The search term for Google Scholar was effects of humor in patients with depression. We searched for recent articles and hence used articles written from 2012 to 2022.

Inclusion Criteria

Original articles in the English language, from 2012 to 2022, related to the objectives of the study; only meta-analytic review articles were included.

Exclusion Criteria

Review (except meta-analytic review) and commentary articles, articles older than 10 years, and articles not written in the English language were excluded.

Results

**Figure 1:**
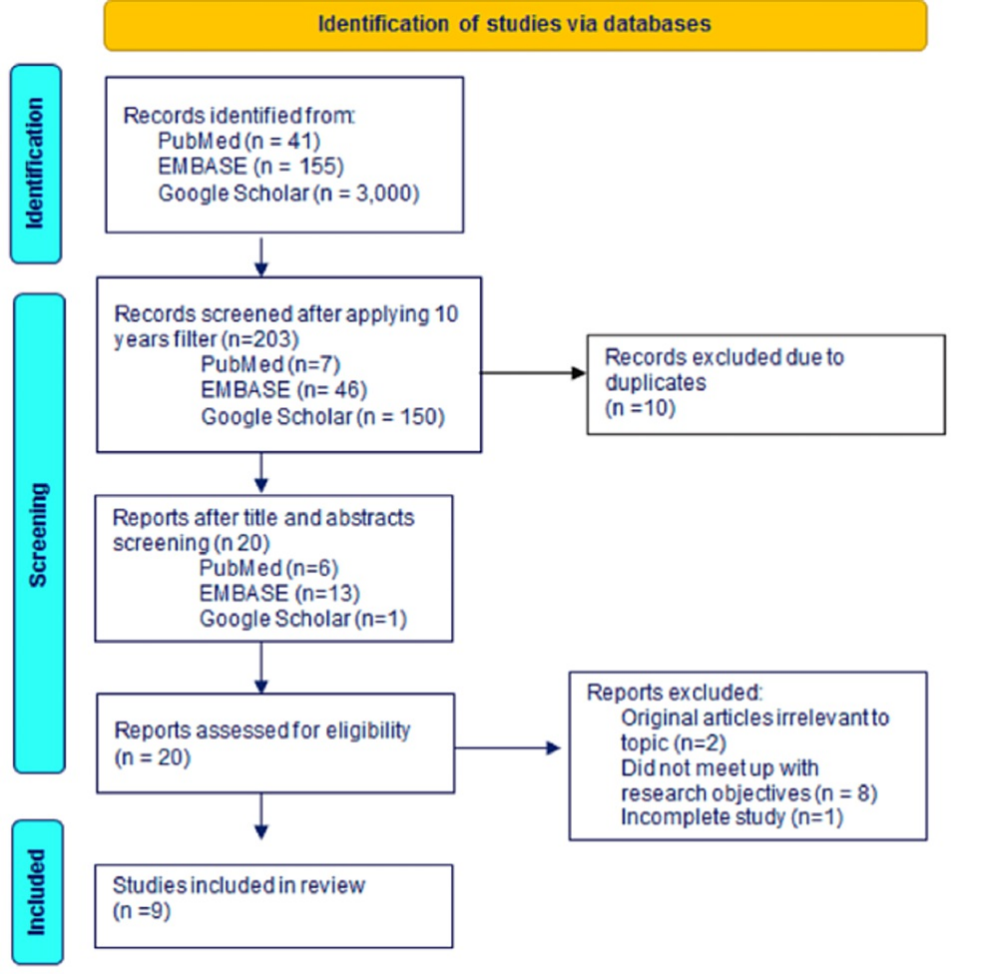
A flow diagram showing the selection process of included articles used in this review

Our data search returned a total of 155,196 articles. Articles were screened for relevance by title which resulted in nine articles (Figure 1). Three of the nine articles were cross-sectional studies, two were correlational studies, one was a semi-randomized study, one was a randomized study, one was a meta-analytic review, and one was an experimental statistical power analysis study. These articles provided a relationship between humor therapies and depression. Humor was found to decrease negative emotions, increase positive emotions, and enhance the distance from adversity. The humor style has also been correlated with depression/suicidal ideation, with the positive style having a positive correlation with depression. It also infers that there is evidence that humor can be a useful tool in managing depression in vulnerable patients.

The Relationship between Humor and Depression

Humor has been used to describe one's state of mind in a general sense or as an attribute of being amusing or comic. Unfortunately, this positive expression in human interaction is deficient, particularly in individuals with mood disorders [5]. This was one aspect of the relationship between humor and depression explored in a study by Berger et al., where he summarized several empirical studies that examined the complex connection between humor and psychopathologies, especially depression [6]. They deduced that the humor abilities of these patients were significantly impaired [6]. In agreement, Falkenberg et al. noted that the humor-abilities of patients with depression were associated with their depression scores, not necessarily impacting their symptoms of depression [7]. One plausible explanation was to look at the neural mechanism of humor from two distinct angles: cognition and emotion, using functional magnetic resonance imaging [6,8]. The cognitive and emotional aspects of humor-processing affect the frontotemporal and mesocorticolimbic brain areas known for language [9] and pleasure [10,11] operations, respectively. Therefore, abnormalities in these brain areas could result in symptoms of depression like social withdrawal and anhedonia [12] thereby underlining the importance of humor.

The Effects of Humor on Patients With Depression

In line with our objective to look at the effect of humor on patients with depression, Konradt et al. studied the effectiveness of humor therapy groups among elderly depressed patients. The two experimental groups were a humor-treatment group and a control group without humor therapy [4]. All participants were diagnosed with depression using the International Classification of Diseases (ICD)-10. They were constantly assessed using questionnaires like the geriatric depression scale, state-trait-cheerfulness inventory, and satisfaction with life scale during pre-treatment and post-treatment. The humor-treatment group had different humor-rich sessions, either by looking at the humorous perspective of everyday things or through conversation, making funny faces, remembering funny things, and so on. Overall, the humor-treatment group showed significant changes in outcomes such as physical health, satisfaction with life, improvement in suicidal ideations, and sad mood [4]. Although the control group also showed improvements in suicidal tendencies and bad moods, which attests to the efficacy of antidepressants, the addition of humor therapy improved quality of life with varied perspectives [4]. Goldberg and Harrow suggested that 'quality of life could predict long-term outcome in patients with depression [13]. Therefore, this would indicate that humor as a form of therapy could improve the long-term outcome of depression.

Congruent with other studies, the meta-analysis by Zhao et al. [14] revealed humor as a therapeutic intervention that relieved depression and improved sleep. The study further highlighted humor as a safe and convenient approach to improving interpersonal relationships [14]. Therefore, it could potentially be an adjunct to psychotherapy and pharmacotherapy. Similarly, a pilot study examined humor's effect on patients' quality of life with late-life depression [15]. The average patients, 78 years of age, all received antidepressants and were further divided into two groups. One group received humor therapy, and the other group had just the standard therapy of antidepressants [15]. The depressive patients who received humor therapy noted better mood, improved depressive symptoms, and higher quality of life [15]. Another study was done to compare the effects of humor, positive reappraisal, and spontaneous emotion regulation on remitted depressed patients and corroborated that humor could be an emotion regulation strategy in coping with stressful events, which could be potential triggers for depression [16]. Humor was found to be as powerful as positive reappraisal as spontaneous regulation of negative emotion in curbing negative feelings and enhancing positive ones [16]. Future studies could pinpoint the form of humor that would be most beneficial and its effects on adapting to depression-triggering situations.

In contrast, a randomized control trial done in Germany investigated the efficacy of humor training among people with depression, anxiety, and adjustment disorder [17]. It reported that outcomes such as depression, anxiety, and well-being were unaffected in the training group. This contradicts existing studies that have substantially shown the benefits of humor as a therapeutic intervention for patients with depression or other mental illnesses [18,19]. According to the study, it is possible that these participants did not commit well enough to the humor training as recommended. Unfortunately, there were no parameters to measure how well participants engaged in the training sessions. This could explain the low to no impact of the humor training on the named outcomes.

The Effect of Humor Styles on Patients With Depression

Studies have shown that the type of humor style expressed could determine the kind of effect experienced. Lee et al. tested the mediating roles of depressive symptoms in adolescents' humor expression and suicidal ideations [20]. The cross-sectional study found that adolescents who consistently used negative humor like 'self-deprecation' (modesty about or criticism of oneself) experienced more depressive emotions and suicidal ideations [20]. At the same time, other types of negative humor like 'other-devaluing' (emphasizing other people's flaws or negative qualities) resulted in less suicidal ideation [20]. This is explained by the 'superiority theory,' which could be traced back to Plato and Aristotle several centuries ago, stating that humor and laughter express feelings of superiority over other people's weaknesses [21]. This in turn helps to relieve tension. Furthermore, Meyer et al. looked at the association between positive ('affiliative' humor helps to improve interactive skills through social bonds, 'self-enhancing' uses humor to cope with stress) and negative ('self-defeating' humor is disparaging oneself for better social interactions, 'aggressive' humor is diminishing others to reinforce oneself) humor styles with suicidal ideations among patients with borderline personality traits [22]. Consistently, it was concluded that positive humor styles mitigated suicidal ideation [22]. However, the 'aggressive' humor style reduced suicidal tendencies among patients with borderline personality traits [22]. Possibly, because aggressive humor helps them cope with negative thoughts about themselves and others; these findings are in line with two other studies done. One was a correlational study, which looked at the effect of the same positive ('affiliative’ & 'self-enhancing') and negative ('aggressive’ & ‘self-defeating') humor styles on psychological health [23]. Psychological health was defined as a healthy and mature state of emotional, behavioral, and social development, using indices such as depression, anxiety, and stress [23]. The second cross-sectional study was done in Israel to explore the dual relationship between humor styles and mental illnesses such as PTSD, depression, and anxiety among terrorism survivors and their spouses [24]. Therefore, both studies concluded that positive humor styles are negatively associated with poor psychological health and vice versa. 

Interestingly, Akram et al. [25] looked at the interpretation of depressive internet memes, which qualify for negative humor, in two groups. One group was diagnosed with depression and the control group was without depression [25]. The study demonstrated that the perception of humor and mood improvement from depressive memes were more among patients with depression than in the control group. Again, this finding points out the benefit of negative humor in patients with depression who often have difficulty verbalizing their feelings. Therefore, depressive memes help them visualize their experience, which in turn gives them emotional and social support as they bond with others. From the above, one could infer that certain types of negative humor do affect depressive symptoms and suicidal ideations, depending on to who the humor is directed. 

Table 1 lists the characteristics and summary of the findings of the articles included in this study.

**Table 1:** The characteristics and summary of findings of articles included for this study

Author/Year	Country of study and characteristics of participants	Effects of humor on depression	Gaps in research	Summary
1. Akram U et al., 2020 [25]	United Kingdom, cross-sectional study design, n=200, mean age = 23.64 ± 10.12, range 18–56, females (74%).	Patients with depression may benefit from negative-style humor memes because they can visualize the experience of depressive symptoms, which in turn helps them form social and emotional bonds theoretically with others.	Predominantly female participants prevent generalization.	The study explores the effects of humorous depressive memes on a depressed patients.
2. Tagalidou N, et al., 2019 [17]	Germany, randomized controlled trial, n=37; predominantly female (n = 27, 73.0%) and had Austrian citizenship (n = 32,86.5%). Age range =24-76 years old with an average age of 50.86	Humor training showed inconsistent results in patients with depression	Larger and more diverse sample size would have positively impacted the study results. Blinding of the participants failed, and the selection was self-selection (selection bias)	This study explores the effects of humor training on depression and how it makes up for the inability to perceive and apply humor in patients with depression and other mental health diseases.
3. Braniecka A et al., 2019 [16]	Poland, experimental statistical power analysis, N=54, 19-60 years of age	Humor was found to decrease negative emotions, increase positive emotions, and enhance better adversity management. Humor curbed negative feelings and enhanced positive feelings among remitted depressed patients	.A larger sample size would increase the statistical power of the study.	Preliminary empirical support for the idea that for individuals vulnerable to depression, humor can be an adaptive tool in dealing with negative responses to aversive events, and thus, it may impair the potential of these events to trigger depressive episodes.
4. Meyer N et al., 2017 [22]	In the United States, a correlational study using the survey method, Bootstrapping analysis, was used; for 176 undergraduate psychology students.	BPD traits were negatively correlated with self-enhancing humor styles and positively correlated with self-defeating ones but were not significantly correlated with affiliative or aggressive humor styles. Affiliative, self-enhancing, and self-defeating humor was found to modify bipolar disorder (BPD) traits and suicidal Ideation (SI).	The study was conducted predominantly on the only female gender and was conducted outside. A study with a more male population would be better to appreciate the effect of an aggressive style of humor	Affiliative, self-enhancing, and self-defeating styles of humor have a modifying effect on BPD traits and SI, while the aggressive style of humor has no modifying effect on BPD traits and SI
5. Konradt B et al., 2012 [4]	Germany, semi-randomized design. Elderly people, 99 patients in total (49 treated and 50 untreated).	Satisfaction with life was significantly improved more in the humor-treated group compared with the non-treated group with P < 0.001. Also, the seriousness of their state, cheerfulness of their state, and physical health were found to improve by p<0.01, p =0.095, and p <0.05, respectively.	Although the study was a semi-randomized design, no follow-up data was obtained after patients discharge from the hospital.	Study findings suggested that humor has a beneficial effect on the standardized treatment of depression. However, there is a need for further study to see the preventative effect of humor and the effect of humor on depression post-discharge,
6. Zhao J et al., 2019 [14]	China, meta-analytic review using the Cochrane guidelines. A total of ten studies and 814 participants are included.	Laughter and humor effectively lower depression in adults with depression, anxiety, and sleep quality.	There is a need for data that reflect the post-follow-up effect of humor and laughter. Post-discharge follow-up data is needed for the long-term effect of the results.	This study collated the impacts of laughter and humor on specific mental disorders like depression, anxiety, and sleep and its effectiveness as an intervention tool.
7. Fatima S et al., 2020 [23]	Pakistan, correlation study design. A sample of 199 (female = 93 and male = 106), university students ages 18 to 26 years, mean 21.02 +/_ 1.78 years	Affiliative humor style was found to have a negative predictor for conditions like stress, anxiety, and depression, while aggressive humor style was found to positively correlate with stress. Furthermore, negative humor styles are negatively correlated with somatic disorders.	The conclusion was not generalizable because of the population sample, which was undergraduate students. The study population need to be more inclusive to be more generalizable	Relationship-oriented positive humor styles are protective against psychological distress, while negative humor styles negatively impact people's physical health.
8. Besser A et al., 2015 [24]	Israel, cross-sectional study. Israeli couples who survived terrorist attacks, n=105 married couples.	The result suggested that benign humor styles were associated with survivors' lower levels of trauma-related symptoms (actor effects) and also had a buffering effect on the spouse (partner effects). More specifically, the use of self-enhancing humor by survivors was negatively associated with spousal symptoms, and the use of affiliative humor by spouses was negatively associated with psychopathology symptoms reported by survivors. For instance, this study shows that aggressive humor is negatively associated with depressive symptoms. Using humor in the face of problems shows resilience and a positive outlook on life, thereby impacting their emotional states.	This study looked at the humor and trauma-related psychopathology among survivors of terror attacks and their spouses. Therefore, it is not generalizable.	This study highlights the role that benign humor may play in coping with traumatic events while considering the dyadic relationships among survivors and their spouses.
9. Lee C-Y et al., 2020 [20]	China, population-based cross-sectional surveys. Public junior high school students with 802 boys and 749 girls.	The type of humor expressed may determine the kind of emotion displayed. This study emphasized the importance of depression and positive emotion in adolescent humor expression and suicidal ideation.	Possible recall bias as questionnaires used was self-reported. The data source used a cross-sectional research design. Therefore, the inference of causality is more conservative.	The adolescents who tended to use self-deprecating humor expressions experienced more depressive emotions and less positive moods and had a higher degree of suicidal ideation.

Strengths and Limitations

The strength of this review was to mostly fulfill the principle of SANRA that was introduced earlier. This review demonstrated its strength in its ability to explore the relationship between the use of specific humor themes and the ability to identify emotional states associated with such themes and how it impacts their depressed state. Across all our selected articles, the positive and negative impacts of humor were demonstrated. Specific studies conducted in clinical settings could have an observer's bias because of the possible influence of the researchers' expectations. Another limitation across the studies reviewed was the attrition effect, as most patients were lost to follow-up. There is a need to use a population size that reflects the distribution of major depressive disorders across race, sex, and social-economic classification. Because of some study designs, the causal relationship between depression and the effect of specific humor types could not be appreciated.

Future research efforts should explore the most adaptive form of humor and its effect on depressive patients or other psychopathologies across different population categories. In addition, there is a need to study the preventive and the post-follow-up discharge effects of humor on depression, thereby creating awareness of humor styles, their benefits, or potential harm. 

## Conclusions

Humor and its styles have revealed the relationships and effects on patients with depression. These studies highlight the impact of humor on depressive symptoms and even everyday psychological stress. Overall, some humor styles have been demonstrated as useful coping tools for depression and possibly improving interpersonal relationships. More research should be conducted with a more diverse population and larger sample sizes to improve generalizability and statistical power, hoping that humor can become a promising adjunctive therapy to conventional psychotherapy.
